# Spectral-Time Multiplexing in FRET Complexes of AgInS_2_/ZnS Quantum Dot and Organic Dyes

**DOI:** 10.3390/nano10081569

**Published:** 2020-08-10

**Authors:** Vera Kuznetsova, Anton Tkach, Sergei Cherevkov, Anastasiia Sokolova, Yulia Gromova, Viktoria Osipova, Mikhail Baranov, Valery Ugolkov, Anatoly Fedorov, Alexander Baranov

**Affiliations:** 1 Faculty of Photonics and Optical Information, ITMO University, St-Petersburg 197101, Russia; 244222@niuitmo.ru (A.T.); s.cherevkov@itmo.ru (S.C.); avsokolova@itmo.ru (A.S.); vaosipova@itmo.ru (V.O.); mbaranov@itmo.ru (M.B.); a_v_fedorov@itmo.ru (A.F.); a_v_baranov@mail.ifmo.ru (A.B.); 2 School of Chemistry, Trinity College, Dublin, Ireland; gromovay@tcd.ie; 3 Institute of Silicate Chemistry of RAS, St-Petersburg 199034, Russia; 193347@itmo.ru

**Keywords:** AgInS_2_, FRET, multiplex analysis, ternary quantum dots, sensing, time-resolved multiplexing, cyanine dyes, nanoparticles

## Abstract

Nowadays, multiplex analysis is very popular, since it allows to detect a large number of biomarkers simultaneously. Traditional multiplex analysis is usually based on changes of photoluminescence (PL) intensity and/or PL band spectral positions in the presence of analytes. Using PL lifetime as an additional parameter might increase the efficiency of multiplex methods. Quantum dots (QDs) can be used as luminescent markers for multiplex analysis. Ternary in-based QDs are a great alternative to the traditional Cd-based one. Ternary QDs possess all advantages of traditional QDs, including tunable photoluminescence in visible range. At the same time ternary QDs do not have Cd-toxicity, and moreover they possess long spectral dependent lifetimes. This allows the use of ternary QDs as a donor for time-resolved multiplex sensing based on Förster resonance energy transfer (FRET). In the present work, we implemented FRET from AgInS_2_/ZnS ternary QDs to cyanine dyes absorbing in different spectral regions of QD luminescence with different lifetimes. As the result, FRET-induced luminescence of dyes differed not only in wavelengths but also in lifetimes of luminescence, which can be used for time-resolved multiplex analysis in biology and medicine.

## 1. Introduction

At present, it is very urgent to develop methods for the simultaneous detection of several analytes using multiplex analysis of biomarkers [1]. One of the most attractive methods is optical identification based on photoluminescence (PL) measurements [2,3,4,5]. PL techniques provide sensitivity down to the single molecule level. Changing of several PL parameters such as PL band spectral position, PL intensity and quantum yield (PL QY), PL lifetime (LT), and emission anisotropy might be used for the detection of analytes [6].

PL multiplexing strategies typically involve the simultaneous use of multiple labels with different PL characteristics: PL band position (spectral multiplexing) and PL lifetimes (LT multiplexing). Spectral multiplexing is the most common method, where number of fluorophores are luminescent in different colors after excitation by light with one or more wavelengths [7,8]. Colloidal semiconductor quantum dots (QDs) are the most attractive fluorescent labels for the multiplex analyses [9]. QDs of different sizes can be excited with light with the same wavelength, which leads to their emission at several wavelengths. These characteristics, combined with high photostability, make them ideal markers for multiplex analyses [10,11,12]. Another promising multiplexing and coding strategy is based on the use of the PL LT of various fluorophores for their differentiation. Time-resolved PL measurements enable to distinguish between different fluorophores with closely matching emission spectra but different PL LT. The LT multiplexing has been realized with organic dyes [13,14], as well as with QD/dye mixtures [15,16]. The most exploring QDs consist of Cd chalcogenides and demonstrate typical PL LTs of several tens of nanoseconds [17]. This PL decay is quite fast and close to the typical decay times of the autoluminescence of the biological environment, which complicates the analysis of the luminescent response of the marker. Also, the presence of toxic Cd (II) restricts the application of these QDs.

To increase and vary the LTs of organic dyes served as luminescent labels, it was proposed to utilize time-resolved Förster resonance energy transfer (TR-FRET) between organic dyes (energy acceptor) and inorganic lanthanide complexes with long LTs (energy donors) [18,19,20,21,22]. In this case, FRET-induced long-lived sensitized luminescence of the labels was analyzed allowing the discrimination of the autoluminescence [23,24,25]. This scheme with long-lived Eu (III) and Tb (III) complexes as energy donors has been realized using the time-gating technique in [23,24,25] where FRET-induced long-lived sensitized luminescence of labels was demonstrated. The FRET-induced prolongation of luminescence LT of acceptor dyes to submicrosecond times has been also shown by an example of donor/acceptor complex with Mn-doped CdS/ZnS QDs as a donor [14].

Ternary QDs (CuInS_2_, AgInS_2_ (AIS), etc.,), reveal PL lifetimes of up to one microsecond [18,19,20,21,22]. The long LTs make ternary QDs promising for LT coding in a donor-acceptor scheme for detecting luminescent labels using TR-FRET [23,24,25,26], which allows one to discriminate short-lived autoluminescence of the biological surroundings and, thus, increase the sensitivity of determinations.

Currently, few studies are devoted to FRET in donor/acceptor systems with ternary QDs as a donor. The FRET from CuInS_2_ QDs to organic rhodamine B has been shown in [27]. The time-gating technique has been used in [28] to demonstrate FRET-induced prolongation of luminescence LT of acceptor in AgInS_2_/ZnS (donor)–CdSe/ZnS (acceptor) QD systems.

Ternary QDs have very wide PL bands with FWHM of several hundred meV, large Stokes shifts, and lifetimes of hundreds of nanoseconds which increase by almost an order of magnitude with changing detection wavelengths from the short-wavelength to the long-wavelength wing of the luminescence band [29]. The latter comes from the existence of a set of exciton energy states inside QDs band-gap, the luminescent transitions from which with different decay times form the broad PL band of t-QDs [30,31]. This fact and large PL bandwidth of ternary QDs allow to potentially realize simultaneously several effective FRET channels from QDs to different acceptors with different absorption and radiation wavelengths, as was recently shown for Mn (II): CdS/ZnS QDs [29] and discussed in [27,32,33]. This yields FRET-induced luminescence of acceptors with different wavelengths and lifetimes, which opens up the possibility of LT coding of the labels.

To the best of our knowledge, until now, ternary QDs have not been used for LT barcoding either as luminescent labels or as donors in FRET systems to expand the range of adjustment of the PL lifetime of organic dyes or analytes detection. In this work, we demonstrate proof-of-concept of spectral-time multiplexing utilizing TR-FRET between AgInS_2_/ZnS QDs and two cyanine dyes (Cy3 and Cy5) and whose absorption bands overlap different regions of the AIS QD PL band having different lifetimes.

## 2. Materials and Methods

### 2.1. Materials

d-penicillamine (99%) and zinc (II) nitrate hexahydrate (99.998%), sodium sulfide nonahydrate (98%) were obtained from Acros Organics (Moscow, Russia); and indium (III) chloride (98%), silver (I) nitrate (≥99.5%), 1-dodecantiol (DDT), 1-octadecene (ODE), oleylamine (OA, 70%), ammonium hydroxide solution (25%), 3-mercaptopropionic acid (MPA, purity > 98%), 2-ethylhexanoic acid (99%), thiourea (99%), triethyleneglycol dimethylether (98%) were purchased from Sigma-Aldrich in Saint Petersburg, Russian Federation. Organic cyanine dyes: 3,3′-diethylthiacarbocyanine iodides (Cy3) and 3,3′-diethylthiadicarbocyanine iodide (Cy5) were purchased from Sigma-Aldrich in Saint Petersburg, Russian Federation. All chemicals were used without further purification. Ultrapure water (Milli-Q) was used throughout the experiments.

#### 2.1.1. Synthesis of AgInS_2_/ZnS QDs

**Synthesis of AgInS_2_ cores:** QDs were synthesized according to the method described by Chen S. et al. [34]. Briefly, 0.017 g of AgNO_3_, 0.0584 g of In(Ac)_3_, 8 mL of DDT, and 210 µL of OA were added to a 25 mL three-necked flask. The resulting solution was degassed under vacuum at a temperature of 135 °C with continuous magnetic stirring for 30 min. Then the flask was heated up to 185 °C under the argon atmosphere for 15 min until the color of the solution turned to bright red. After the synthesis was completed, 4 mL of ODE was injected into the flask, and the reaction solution was heated up to 210 °C and was immediately used for the further first shell growing that is described below.

**ZnS Shell Growing:** the ZnS shell was formed in two steps.

The first ZnS shell layering procedure was carried out as follows. Zn precursor was prepared by mixing 4 mL of ODE and 0.203 g of zinc stearate in a two-necked flask. The resulting solution was degassed under vacuum at a temperature of 120 °C with constant magnetic stirring for 30 min. The flask was heated up to 160 °C under inert atmosphere of argon for 20 min until the zinc stearate was completely dissolved. Total of 2.5 mL of the obtained Zn precursor was injected into a three-necked flask with the reaction mixture of AgInS_2_ cores from the previous section. Then 0.5 mL of zinc precursor was injected every 15 min. In total, the solution was heated with stirring for 45 min at 210 °C.

After the first shell synthesis was completed, the reaction solution was cooled to the room temperature. An excess amount of hexane was added to the resulting solution. Small portions of acetone were added to the solution, after which portions of particles precipitated according to their size. All fractions of precipitate from green color to orange were redissolved in toluene.

The second ZnS shell layer was applied using the procedure from [35]. The zinc and sulfur precursors for second shell growth were prepared according to the following procedure. Zinc oxide (30 mmol), 2-ethylhexanoic acid (61.5 mmol) (99%), and 40 mL of ODE were mixed, the reaction mixture was heated to 120 °C, and kept until clear solution formed under continuous stirring. The sulfur precursor was prepared by dissolving 34 mmol of thiourea (99%) in 40 mL of triethyleneglycol dimethylether (98%) under sonication.

For the second ZnS shell growth the earlier obtained solution of green QDs in toluene was mixed with 12 mL of ODE and 12 mL of OA in a three-neck flask. The reaction mixture was kept under vacuum at 100 °C for 25 min. Then, 5 mmol of the zinc precursor and 5 mmol of the sulfur precursor were added dropwise to the solution at 180 °C within 1 h. The QDs were purified using a procedure similar to that used for the cores.

#### 2.1.2. Solubilization of AgInS_2_/ZnS QDs

**Figure 1:**
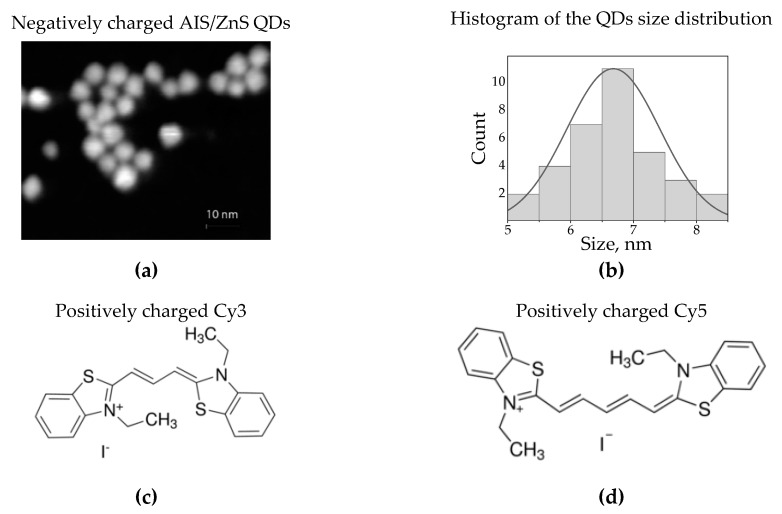
(**a**) Typical TEM image of synthesized water soluble AIS/ZnS quantum dots (QDs) with mean diameter of 6.7 nm; (**b**) histogram of the QDs size distribution; (**c**,**d**) are the structural formulas of the Cy3 and Cy5 molecules, respectively.

The QDs were solubilized by the procedure described in [35]. A portion of the QD solution was purified twice using methyl acetate and hexane as the precipitant and the solvent, respectively. After the second precipitation, the QDs were dissolved in 0.8 mL of chloroform, 0.2 mL of a 15 mg/mL cysteine hydrochloride (98%) solution in methanol was added, and the mixture was stirred for 5 min. The QDs were purified twice using methanol. This procedure was repeated twice. The precipitate was dissolved in 0.65 mL of a 0.01 M aqueous solution of KOH. A concentration of QDs in used stock solution was determined gravimetrically to 2.5 × 10^−7^ M. The Scanning Transmission Electron Microcope (STEM) images of synthesized water-soluble AIS QDs shown in Figure 1 a revealed the approximately spherical shape of the QDs with an average size of 6.7 ± 0.8 nm.

#### 2.1.3. Formation of QD/Cy Donor/Acceptor Complex

The structural formula of the Cy3 (3,3′-diethylthiacarbocyanine iodide) and Cy5 (3,3′-diethylthiadicarbocyanine iodide) molecules used for complex formation are presented in Figure 1b,c, respectively. The concentration of both dyes in the stock aqueous solutions was C = 10^−6^ M. Aqueous solutions of negatively charged AIS QDs were mixed with positively charged cyanine molecules of Cy3 or Cy5. A coulomb electrostatic interaction of the oppositely charged cysteine capping AIS QDs and cyanine molecules results in the formation QD/dye complexes. Similar principle of complex formation was utilized in [36,37,38]. To examine the FRET effect, the PL intensities of QDs and dyes have been analyzed as a function of the ratio of the QD/dye concentration in the mixture solution. QD concentration (*C_QD_*) remained constant while the dye concentrations (*C_dye_*) varied. To prepare the samples with the molar ratio of dye/QD varying from 0.3/1 to 3/1, the drops of cyanine solution were added to the 2 mL QD solution (*C_QD_* = 2.5 × 10^−7^ M). To avoid PL reabsorption the concentration of QDs in the QD/dye mixtures was kept below 2.5 × 10^−7^ M. The absorption and PL spectra of the mixture samples were measured after each stage of dye addition. All optical measurements were performed at room temperature within four hours after preparation of the mixture of solutions.

### 2.2. Equipment

The UV-Vis absorption spectra were recorded using a UV-Probe 3600 spectrophotometer (Shimadzu, Kyoto, Japan). The steady-state PL and photoluminescence excitation (PLE) spectra were obtained with Cary Eclipse spectrofluorometer (Agilent Santa Clara, CA, USA). Time-resolved PL measurements were performed using a time-correlated single photon counting (TCSPC) fluorescence microscope MicroTime 100 (PicoQuant, Inc., Berlin, Germany) equipped with a 405 nm pulsed laser LDH-P-C-405B (PicoQuant, Inc., Berlin, Germany) with pulse duration of 20 ps.

The PL for time-resolved PL measurements was collected by a single photon PMT detector in the spectral range of 430–780 nm. If necessary, a holographic bandpass filter with a bandwidth of 10 nm, tunable in the spectral range of 430–780 nm, was used to select the detection wavelength. The minimum pulse rate of the MicroTime 100 system of 2.5 MHz (corresponding to a 400 ns time range between two laser pulses) was shifted down to a 500 kHz laser pulse rate (corresponding to 2 µs) for measurements of long PL lifetimes. Laser frequency was reduced by implementing an external synchronization using an additional signal generator SFG-71003 (Good Will Instek, Montclair, CA, USA). STEM images of the AgInS_2_/ZnS QDs were measured with a Scanning Electron Microscope Merlin (Zeiss, Oberkochen, Germany) operated at 30 kV on copper grids with ultrathin carbon films. The thermogravimetric analysis (TGA) was performed on a STA 429 CD Simultaneous thermal analyzer (Netzsch-Gerätebau GmbH, Germany) using a platinum–platinum rhodium holder for TG + DSC samples. All optical measurements have been performed at standard ambient conditions three to five times. The errors were calculated as the deviation from the mean value, determined from the set of measurements.

## 3. Results

**Figure 2:**
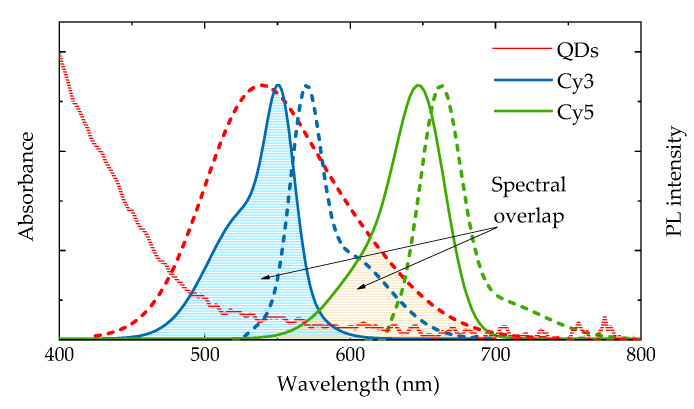
Absorption (red solid line) and photoluminescence (PL) (red dotted line) spectra of aqueous solutions of AgInS_2_/ZnS QDs as well as the normalized absorption (blue and green solid lines) and PL (blue and green dotted lines) spectra of Cy3 and Cy5 dyes, respectively. PL excitation: QDs–405 nm, Cy3–520 nm and Cy5–620 nm, respectively.

Figure 2 shows the UV-Vis and PL spectra of water-soluble AgInS_2_/ZnS QDs (AIS QDs) with an average diameter of 6.7 nm as well as the normalized UV-Vis and PL spectra of Cy3 and Cy5 dyes. Absorption of AIS QDs represents unstructured spectra without excitonic peak typical for ternary QDs [39]. The AIS QD PL band has maximum at 540 nm, a full width at half maximum (FWHM) of 110 nm (450 meV). PL quantum yield is 5.6%. The low energy absorption (PL) bands of Cy3 and Cy5 cyanines have maxima at 550 (570) nm and 647 (663) nm, respectively. It is seen from Figure 2 that the spectral overlap of the donor (QD) PL with acceptors (Cy3 or Cy5) absorption bands takes place in both cases that allow FRET from QD to molecules.

Here and further, unless otherwise specified, a 405 nm radiation was used for excitation of PL of AgInS_2_/ZnS QDs while radiations of 520 nm and 620 nm were used for excitation of the Cy3 and Cy5 PL, respectively. The 520 nm and 620 nm radiations allow direct excitation of the cyanines, since at these wavelengths there are strong cyanine absorption bands, while the QDs absorption is negligible. Contrarily, the 405 nm emission excites solely QDs since there is a local minimum of the both cyanines absorption at this wavelength. This approach makes it easy to control changes in the PL intensity and lifetime both for QD and dyes and evaluate the efficiency of energy transfer in the QD/dye complexes.

To evaluate the theoretical FRET efficiency for the studied QD/dye donor/acceptor pairs, the Förster radius was calculated for the considering QD/dye pairs from the following equation:

(1) $$R_{0}=9780\;{\left(k^{2}\;n^{-4}\;\cdot QY_{D\;}\cdot \int F_{D}\;\left(\lambda \right)\;\varepsilon_{A}\;(\lambda )\lambda^{4}d\lambda \right)}^{1/6},$$

where *κ*^2^ is the orientation factor of the transition dipole moments (typically, *κ*^2^ = 2/3), *n* is the refractive index of medium, *QY_D_* is the quantum yield of QDs, *F_D_*(*λ*) is the normalized PL spectrum of the QD, *ε_A_*(*λ*) is extinction of the dye at *λ*. The Förster radii for pairs of QDs with Cy3 and Cy5 were estimated as *R*_0_ = 4.05 nm and 4.12 nm, respectively. The theoretical efficiency of FRET was calculated according to the formula:

(2) $$E_{FRET}=\frac{R_{0}^{6}}{R_{0}^{6}+R^{6}}$$

where *R* = 3.4 nm is the QD radius and the size of the ligand *R_L_* = 0.5 nm. Equation (2) gives 60% and 57% for Cy3 and Cy5, respectively. The values of the quantities in Equation (1) are shown in Table 1.

**Figure 3:**
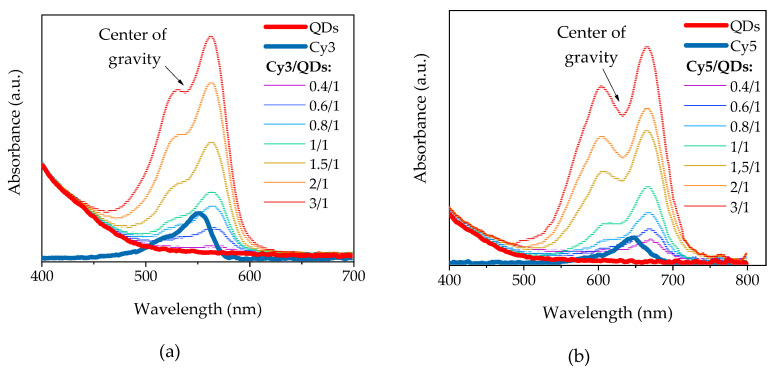

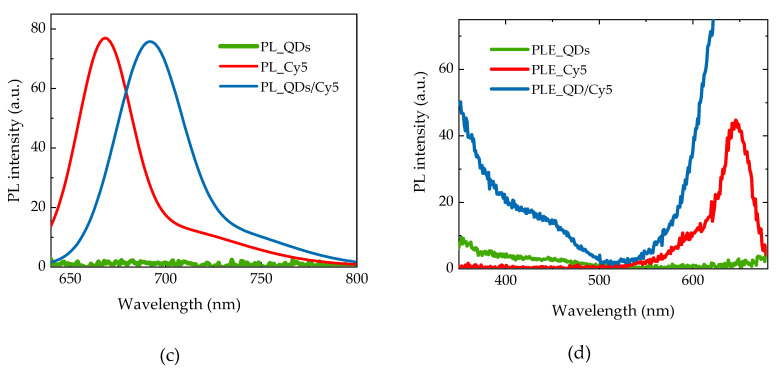
Absorption spectra of mixing solutions of AIS QDs and Cy3 (**a**) and Cy5 (**b**) at different molar rations of dyes and QDs (*n* = *C_dye_/C_QDs_*); (**c**) PL spectra of QDs and Cy5 in solutions, and Cy5 in the complex with QDs, excitation at 620 nm; (**d**) PL excitation spectra (EPL) of QDs and Cy5 in solutions, and Cy5 in the complex with QDs. Detection wavelength of 700 nm corresponds to peak of the Cy5 PL in the complex.

Mixing the aqueous solutions of oppositely charged QDs and molecules of Cy3 or Cy5 results in the formation of QD/dye complexes. The signature of the complex formation is 25 nm red shift of the low energy absorption band of both dyes as compared with those of the free molecules in solution that is shown in Figure 3a,b. Similar red shifts of the dye PL bands are also observed that are illustrated in Figure 3c for Cy5 as an example. It should be noted that the increasing of the dye concentration causes the appearance of the new band in the blue region of dye absorption spectra, indicating the formation of the dye dimers. Similar effects were observed in the reports [40,41]. An additional clear evidence of complex formation is FRET from QDs to dye molecule reflecting in the appearance the absorption spectra of QDs (donor) in the PL excitation spectra of cyanines (acceptor). This is illustrated in Figure 3d for Cy5/QD complexes as an example.

**Figure 4:**
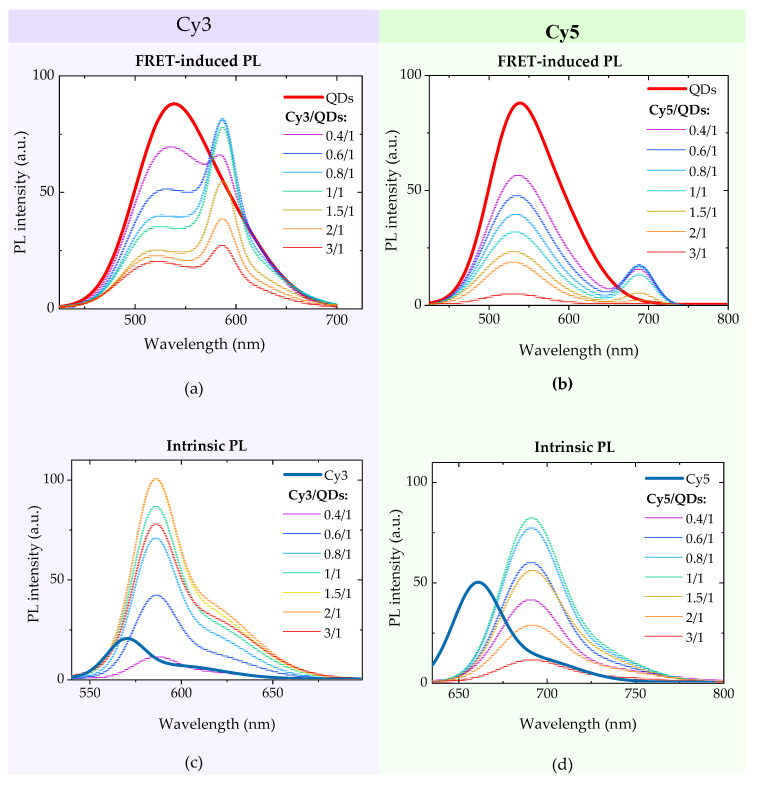

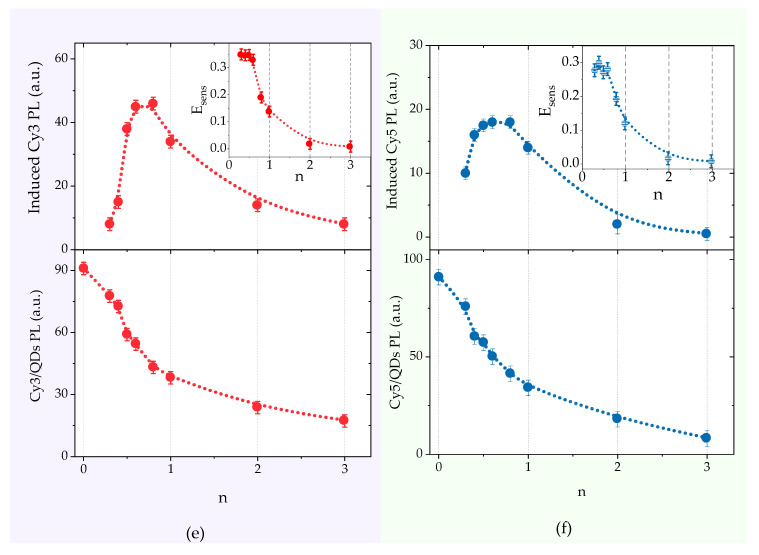
(**a**–**d**) PL spectra of AIS QDs and cyanine dye complexes at different molar ratio of dyes and QDs, *n = C_dye_/C_QD_*. (**a**) Förster resonance energy transfer (FRET)-induced PL spectra at 405 nm excitation of QDs/Cy3 complexes; (**b**) FRET-induced PL spectra at 405 nm excitation of QDs/Cy5 complexes; (**c**) intrinsic PL at 520 nm excitation of QDs/Cy3 complexes; (**d**) intrinsic PL at 620 nm excitation of QDs/Cy5 complexes; (**e**,**f**) intensity of FRET-induced PL of acceptor (Cy3/Cy5) at 405 nm excitation as a function *n = C_dye_/C_QD_* (top), donor (QDs) quenching in the presence of acceptor (Cy3/Cy5) (bottom)**,** FRET efficiency, estimated using Equation (4) for complexes QDs/Cy3 andQDs/Cy5, respectively (top, inset).

The experimental efficiency of FRET can be estimated using the efficiency of sensitization of the PL acceptor *E_sens_*. A 405 nm radiation was used for selective excitation of the AgInS_2_/ZnS QDs, where dyes do not absorb the light, while the 520 nm and 620 nm radiations allowed direct excitation of the Cy3 and Cy5 PL, respectively, as the QD excitation at these wavelengths was negligible. Therefore, an appearance of dye PL in the QD/dye complexes at 405 nm excitation, where absorption of dyes was negligible, indicates FRET from QDs to dyes. This approach makes it easy to control changes in the PL intensity and lifetime both for QDs and dyes and evaluate the efficiency of energy transfer in the QD/dye complexes. The fraction of energy that goes into the excitation of a sensitized PL acceptor, E_sens_, can be estimated from experimental data by using the following equation:

(3) $$E_{sens}=\frac{I_{AD}\;\left(\lambda_{D}^{ex}\right)/D_{QD}\;\left(\lambda_{D}^{ex}\right)}{I_{A}\;\left(\lambda_{A}^{ex}\right)/D_{A}\;\left(\lambda_{A}^{ex}\right)}$$

where *D_A_* and *D_QD_* are the optical densities of the acceptor and donor at the excitation wavelengths of the PL; *I_AD_* is the intensity of sensitized acceptor PL, *I_A_* is the intensity of acceptor PL directly excited by light; *λ_D_* and *λ_A_* are the wavelengths of the selectively exciting PL. F is the efficiency of donor PL quenching:

(4) $$F=1-\frac{I}{I_{0}},$$

where *I* and *I_0_* are the donor PL intensities in presence and in absence of the energy acceptor, respectively. Figure 4a,b illustrates the PL spectra of AIS QDs and FRET-induced PL of cyanine dyes as a function of molar ratio of dyes and QDs, *n = C_dye_/C_QD_*. The mixtures are excited by radiation with a wavelength of 405 nm. The same dependence of intrinsic PL of dyes in QD/dye complexes is presented in Figure 4c,d. PL spectra of Cy3 and Cy5 in complexes were excited at 520 nm and 620 nm, where QDs do not absorb the light.

As can be seen from Figure 4a,b, as well as from Figure 4e,f, the intensity of sensitized PL of both dyes increases with dye molar concentration, reach a maximum at *n* about (0.7–1/1) and then begins to decrease. The QD PL intensity decreases with *n* because of quenching. The saturation and following reduction of PL intensity of studied dyes in complexes with increasing *n* was also observed at dyes direct excitation by 520 nm and 620 nm radiations, respectively, as shown in Figure 4c,d, but never in diluted solutions of free dyes. The reduction of the dyes PL intensity indicates, most likely, the formation of non-radiative dye dimers that can form at the QD surface. During the formation of dimers, the ratio of the intensities of the first and second absorbance peaks of the dye increases in comparison with the monomeric form [32,41]. These changes in the ratio of absorbance bands can be observed for both Cy3 and Cy5 in Figure 3a,b, which is the evidence of dimer formation [42]. As a result, part of energy passed by FRET from QD-donor to dye-acceptor is lost as a result of non-radiative relaxation.

To minimize the effects of the dye aggregation in further TR-FRET expriments we limited ourself by QD/dye complexes with *n* = *C_dye_/C_QD_* = 1/1.

### Time-Resolved FRET

**Figure 5:**
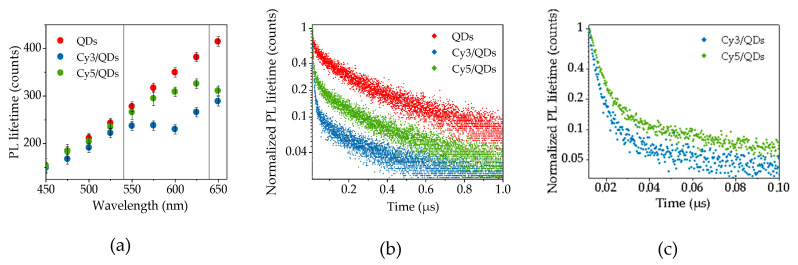
(**a**) The dependence of average PL lifetimes (‹τ›) on detection wavelengths; the vertical black lines indicate the gravity centers of the dye absorption; (**b**) the decay curves at *λ_exc_* = 405 nm, *λ_det_* = 625 nm of AIS QDs (red) and AIS QDs in complex with Cy3 (blue) and Cy5 (green); (**c**) the decay curves of QD/Cy3 (green) and QD/Cy5 (blue), *λ_exc_* = 405 nm, *λ_det_* = 585 nm for Cy3 and *λ_det_* = 700 nm for Cy5 (the wavelengths of dye PL maxima).

The studied AIS QDs demonstrate long PL lifetimes with little increase in PL wavelength (Figure 5a). Similar dependences were observed in the work of [21,43]. The AIS QDs PL decay curves measured at different detection wavelengths were well fitted by two exponents:

(5) $$I\left(t\right)=A_{1}e^{-\frac{t}{\tau 1}}+A_{2}e^{-\frac{t}{\tau 2}}\;$$

with average PL lifetimes calculated by the following equation:

(6) $$‹\tau ›=\frac{A_{1}\tau_{1}^{2}+A_{2}\tau_{2}^{2}}{A_{1}\tau_{1}+A_{2}\tau_{2}}$$

where *A*_1_ and *A*_2_ are the amplitudes and *τ*_1_ and *τ*_2_ are the decay times of first and second exponent. An average AIS QD PL lifetime dependence on detection wavelengths presented in Figure 5a shows that *‹τ›* values varied from ~150 ns at 450 nm to ~415 ns at 650 nm.

Comparison of Figure 2; Figure 5a revealed that the dye absorption bands overlap different spectral regions of the AIS QD PL band having different lifetimes. For simplicity of estimates, we assume that the absorption bands of Cy3 and Cy5 molecules are at ~540 nm and ~640 nm where the average QD PL lifetimes are ~255 ns and ~400 ns, respectively. Then, we can expect different decay times for FRET-induced PL of Cy3 and Cy5 dyes.

FRET in the complexes results in the decreasing of QD PL lifetimes as can be observed in Figure 5a. The reduction of the QD PL lifetimes is spectrally dependent. The PL lifetimes of QDs in the complexes with Cy3 begin to decrease markedly in spectral region of Cy3 absorption at 500–520 nm (Figure 2), while QD/Cy5 PL lifetimes were close to free QDs at this spectral area and start to decrease at the wavelengths of 575–600 nm, where Cy5 absorbs the light. This behavior is consistent with the model in which AIS QDs have a few of in-bandgap energy sublevels with lifetimes increasing with reduction of their energy. These energy sublevels are formed because of the structural and surface defects of QDs [43,44,45,46,47,48]. Then each sublevel can be considered as an initial energy state of donor for the FRET to the acceptor. FRET from the QD to the dye occurs with higher probability from the sublevel that is closer to the dye one in energy.

Figure 5a,b presents PL lifetime of AIS QDs detected at *λ_det_* = 625 nm in solution (red) and in complexes with Cy3 (blue) and Cy5 (green) upon excitation with *λ_exc_* = 405 nm, showing a decrease in average PL lifetimes of QDs from 380 ns to 260 ns and 325 ns for Cy3 and Cy5, respectively. This fact supports FRET from QDs to dyes. The decays of FRET-induced PL of dyes in complexes with QDs were measured at 585 and 700 nm for Cy3 and Cy5, respectively, upon excitation of 405 nm. These PL decay curves are represented in Figure 5c. Since the PL of Cy3 is superimposed with the QD PL, the PL of QD/Cy3 complexes at the wavelength of 585 nm was fitted into three exponents: two exponents related to QDs, as described above, and one exponent to dye. The PL decay of Cy5 dye was well approximated by one exponent. We found that the lifetimes of FRET-induced PL of Cy3 and Cy5 dyes significantly increased by more than the order of magnitude from 0.6 ns and 0.5 ns to 5 ns and 9 ns, respectively, as compared with those in free form. The marked difference in FRET-induced PL decay times between Cy3 and Cy5 allows to make reliable selection between two dyes by using simple time-gate detection technique. The difference in spectral positions and lifetimes of the FRET-induced PL of two dyes-acceptors demonstrates spectral-time multiplexing by using AIS QDs as a donor.

In the present study the Cy3 and Cy5 cyanine dyes were chosen as a model objects since their absorption bands overlap the QD PL at different spectral areas (540 nm and 640 nm, respectively) possessing different PL lifetimes (~255 ns and ~400 ns, respectively). For real biomedical applications, the appropriate luminescent dye-labels differing in spectral positions of their absorption bands should be selected and the problems of low FRET efficiency and dye PL quenching due to aggregation on the QD surface must be overcome.

## 4. Conclusions

In present proof-of-concept study we have demonstrated on the model objects Cy3 and Cy5 cyanine dyes the difference in spectral positions and lifetimes of the FRET-induced PL of two dyes-acceptors using AIS QDs as a donor, which makes it possible to use this system for time-resolved multiplex analysis. We have shown that in complexes of AIS QDs with Cy3 and Cy5 cyanine dyes an effective FRET occurred with a significant increase in the intensity and lifetime of dye PL. The absorption bands of Cy3 and Cy5 overlap the QD PL band at different spectral areas (540 nm and 640 nm, respectively), possessing different QD PL lifetimes (~255 ns and ~400 ns, respectively), since the PL lifetimes of AIS QDs are spectrally dependent. As a result, FRET-induced PL of Cy3 and Cy5 have different lifetime of 5 and 9 ns at 585 nm and 700 nm, respectively. It was also shown that with an increase in the ratio of dye to QD, and consequently the average amount of dye molecules on one QD, the total quantum yield of the dye as well as FRET efficiency decreased because of dye aggregation and self-quenching. In the present study, we have determined the optimal dye to QD ratio for the highest dye FRET-induced PL. The results of this study can be used to create new advanced method for time-resolved multiplex analysis, which can be applied in various fields of biology and medicine, including sensing and enzyme immunoassay.

## Figures and Tables

**Table 1:** The values of the quantities for Equation (1).

Complex	Extinction of the Dye (*ε_A_*)	Quantum Yield of QDs (*QY_D_*)	Overlap Integral
QDs/Cy3	140,000	5.6%	4.7 × 10^−13^
QDs/Cy5	230,000	5.6%	4.2 × 10^−13^
